# Bilateral breast cancer in China: A 10‐year single‐center retrospective study (2006–2016)

**DOI:** 10.1002/cam4.4141

**Published:** 2021-08-04

**Authors:** Hanfang Jiang, Ruyan Zhang, Xiaoran Liu, Ran Ran, Jiayang Zhang, Yaxin Liu, Xinyu Gui, Yifei Chen, Kun Li, Bin Shao, Ying Yan, Xu Liang, Guohong Song, Lijun Di, Huiping Li

**Affiliations:** ^1^ Key Laboratory of Carcinogenesis and Translational Research (Ministry of Education/Beijing), Department of Breast Oncology Peking University Cancer Hospital & Institute Beijing China

**Keywords:** bilateral breast cancer, metachronous, prognosis, proportion, synchronous

## Abstract

Bilateral breast cancer (BBC) is an uncommon subset of breast cancer (BC), and it may present as synchronous bilateral breast cancer (sBBC) or metachronous bilateral breast cancer (mBBC). Through this study, we aimed to evaluate the proportion of BBC in BC and compare the clinicopathological characteristics, treatment, and outcomes of sBBC and mBBC at an academic cancer center in China. Patients with BC consecutively treated between 2006 and 2016 were retrospectively reviewed. Patients with BBC were included. In total, 3924 patients with BC were analyzed and 127 patients with BBC (28 sBBC, 99 mBBC) with a median follow‐up of 98 months were identified. The proportion of BBC was 3.2% (0.7%, sBBC; 2.5%, mBBC). The median age at the first diagnosis of mBBC was significantly younger than that at the first diagnosis of sBBC (*p* = 0.027). Patients diagnosed as having sBBC were more likely to have a positive family history (*p* = 0.047). The first tumors of mBBC were detected at a significantly earlier tumor stage compared with those of sBBC (*p* = 0.028). The concordance rates of histopathologic type in the first and second tumors were 60.7% and 58.0% in sBBC and mBBC, respectively. sBBC had a significantly poorer disease‐free survival than mBBC did (*p* = 0.001). BBC is a rare disease affecting the Chinese population. sBBC is associated with a greater prevalence of a family history of breast cancer and poorer prognosis, compared with mBBC.

## 1 INTRODUCTION

Breast cancer (BC) is the most commonly diagnosed cancer and the leading cause of cancer‐related deaths among women worldwide.^1^ In China, BC is the most common tumor in women, and it was the fifth most common cancer causing cancer‐related deaths in 2015.^2^ Bilateral breast cancer (BBC), defined as the presence of primary cancer in each breast, is an uncommon subset of BC. The incidence of BBC has increased in recent years, ranging from 1.4% to 11.8% in Western countries.^3^, ^4^, ^5^, ^6^ In the Asian population, the incidence of BBC ranges from 2.8% to 3.2%.^7^, ^8^, ^9^ However, limited data exist about the incidence of BBC in the Chinese population within the last 10 years.

BBC is further divided into synchronous bilateral breast cancer (sBBC) and metachronous bilateral breast cancer (mBBC). Younger age^5^, ^10^, ^11^, ^12^ and family history^13^, ^14^, ^15^ are the risk factors for BBC. Diaz *et al*.^10^ reported a median age of 51 years for mBBC and 71 years for sBBC in Spain, whereas O’Brien *et al*.^16^ reported a median age of 52 years for mBBC and 59 years for sBBC. A large sample of 4403 cases of sBBC and 7159 cases of mBBC from the surveillance, epidemiology, and end results program (1998–2011) in the United States showed an average age of 59.4 years for mBBC and 63.1 years for sBBC.^17^ Verkooijen *et al*.^18^ have reported that young women have a higher risk of developing mBBC, whereas older women have an increased risk of developing sBBC. In China, the median age at diagnosis of women with BC was almost 10 years younger than that of Western Caucasian patients^19^; however, few studies have focused on the age of onset of BBC in the last 10 years, and sufficient data in this regard are lacking. Therefore, more research on the age of onset of BBC in Chinese women is warranted.

It is generally believed that the survival rate of mBBC is higher than that of sBBC; however, there is no significant difference between the survival rates of mBBC and unilateral BC.^20^, ^21^, ^22^ Baretta *et al*.^17^ have reported that among patients with sBBC, those with inconsistent bilateral estrogen receptor (ER) status have worse prognosis than those with bilateral ER all‐positive status do, and patients with bilateral ER all‐negative status have the worst prognosis. Patients with BBC have an increased risk of developing non‐BC primary cancer and are more likely to die from it.^23^ sBBC has been reported to be an independent risk factor for survival outcomes and distant metastases.^4^, ^24^ Sim *et al*. have reported that sBBC has a significantly poorer overall survival (OS) than mBBC does.^9^ Therefore, early identification of patients with BBC is important for timely prevention and treatment, and improvement of prognosis.

Some studies on Chinese women with BBC have been reported; however, these are either very old or they focus on aspects such as prognostic factors, or mBBC is defined as the development of two tumors over more than 12 months in these studies.^25^, ^26^, ^27^ This study aimed to evaluate the proportion of BBC in BC and analyze the clinicopathological characteristics, treatment, and outcomes of sBBC and mBBC between 2006 and 2016 at the Peking University Cancer Hospital, which is an academic cancer center in China.

## 2 MATERIALS AND METHODS

### 2.1 Study design and data source

A total of 3924 women with BC who were consecutively treated at the Department of Breast Oncology of the Peking University Cancer Hospital between 2006 and 2016 were retrospectively reviewed. This study was approved by the Ethics Committee of Peking University Cancer Hospital (Approval ID: 2020YJZ82), which waived the requirement for patient signed informed consent owing to the retrospective nature of the study. And all procedures performed in this study involving human participants were in accordance with the ethical standards of the Ethics Committee of the Peking University Cancer Hospital and with the declaration of Helsinki. Patients with BBC were identified according to the criteria described by Chaudary *et al*.^11^ Briefly, BBC included tumors in situ of the contralateral breast, the second tumor with a different histological type or higher histological differentiation level than that of the first breast lesion, and there was no evidence whether the cancer was local, regional, or distant metastasis. According to the 6‐month interval of contralateral BC, BBC was further categorized as sBBC and mBBC.^28^ Patients with stage IV first BC and those who were found to have distant metastases between the first and second primary BC were excluded, unless the two BCs had different histopathological types.

Patient demographics (age at first diagnosis of BC, menopausal status, family history of BC, and time interval between the first and second primary breast tumors), tumor characteristics [size, tumor stage, histopathology, ER, progesterone receptor (PR), and human epidermal growth factor receptor 2 (HER2) status], axillary node status, and treatments (surgery for the first BC, radiation therapy, systemic chemotherapy, and endocrine therapy for the first BC) were recorded. The tumor stage was determined using the TNM classification of AJCC 7^th^ edition.^29^ To compare the clinicopathological characteristics of the first tumor between patients with sBBC and patients with mBBC, the first tumor of sBBC with identical diagnosis dates was defined to the tumor with the more advanced stage, or the larger size if the same stage, or the higher grade if the same size.^18^, ^30^ In case of similar tumor sizes, the higher grade tumor was considered as the first tumor. A family history of BC was defined as having at least a first‐ or second‐degree relative with this disease.

### 2.2 Statistical analysis

Differences in categorical data between the groups were tested using the Chi‐square test, and Fisher's exact test was used where the expected numbers of the patients were fewer than five. Continuous variables were tested using Student's *t*‐test. Differences between the medians of continuous variables were tested using the Mann–Whitney *U* test. Survival studies were performed from the date of diagnosis of the first cancer. Kaplan–Meier survival analysis was used to determine the disease‐free survival (DFS) rates. The statistical significance of the differences in survival between the groups was determined by the log‐rank test. All statistical tests were two sided. Statistical significance was defined as *p* < 0.05. Statistical analyses were conducted using the software package SPSS version 20.0 (SPSS, Inc.).

## 3 RESULTS

**FIGURE 1:**
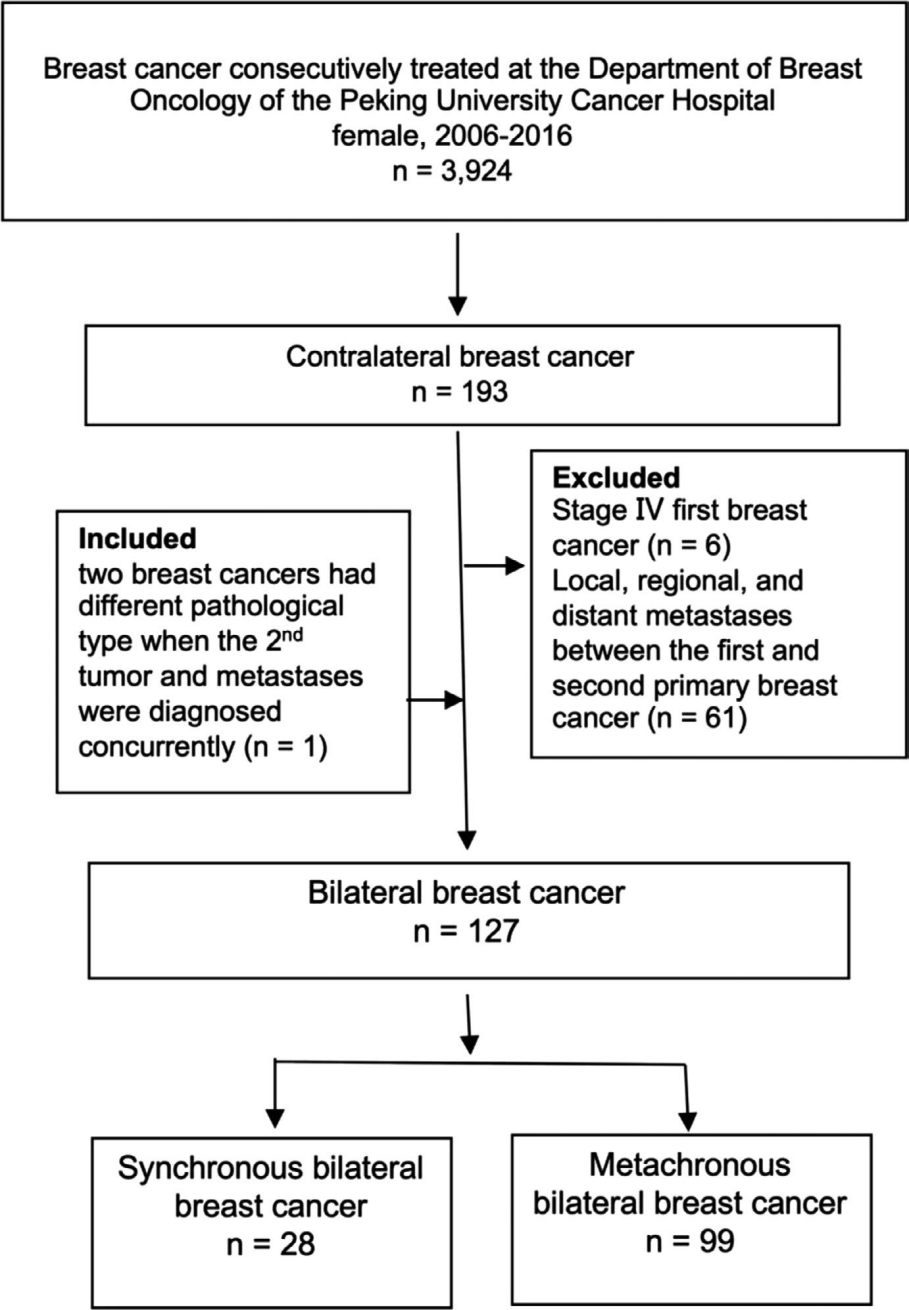
Flow diagram illustrating the identification of patients with bilateral breast cancer in this study

**FIGURE 2:**
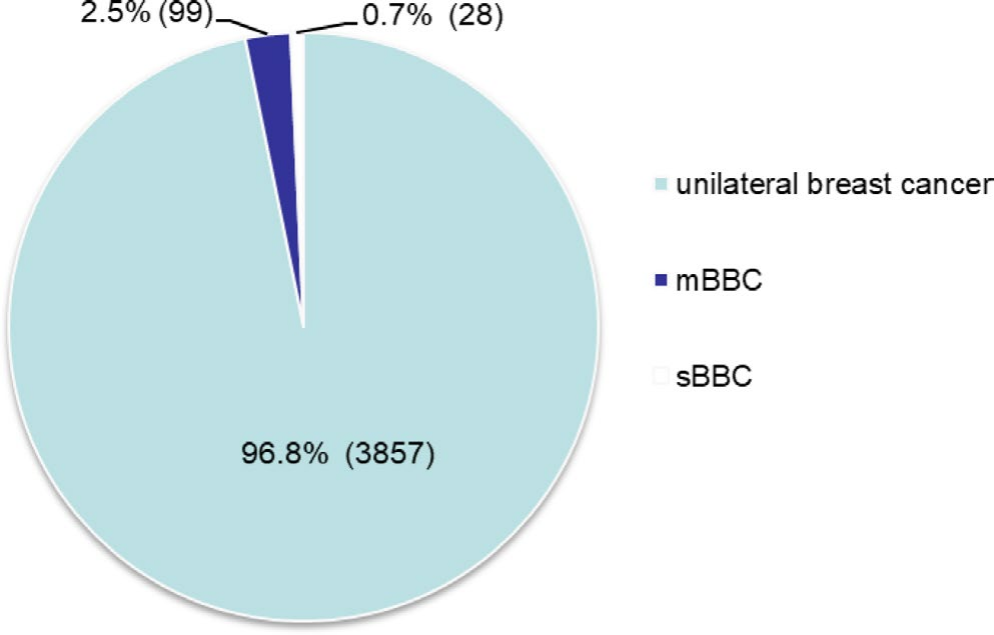
Proportion of synchronous bilateral breast cancer and metachronous breast cancer among patients with breast cancer between 2006 and 2016. mBBC, metachronous bilateral breast cancer; sBBC, synchronous bilateral breast cancer

A total of 127 patients were finally enrolled in the study. The identification of patients with BBC is illustrated in Figure 1. The proportion of BBC was 3.2% (127 of 3924), including 2.5% (99 of 3924) of mBBC and 0.7% (28 of 3924) of sBBC (Figure 2).

### 3.1 Patient characteristics

The characteristics of the patients are listed in Table 1. The overall median age of the patients with BBC was 45 years. The median age of the patients at the first tumor diagnosis of mBBC was significantly younger than that at the first diagnosis of sBBC (median age: 44 vs. 53.5 years, *p* = 0.027), and 77.8% of patients with mBBC and 46.4% of patients with sBBC were pre‐menopausal. The median time interval between the first and second tumors was 68 months, with a mean duration of 85.2 ± 6.7 months among patients with mBBC, and 80.8% of the second tumors (80/99) were diagnosed within 10 years of the diagnosis of the first tumor.

**TABLE 1:** Characteristics of patients with synchronous bilateral breast cancer compared with metachronous breast cancer

Characteristic	Synchronous bilateral breast cancer (n = 28)		Metachronous bilateral breast cancer (n = 99)		*p* value^a^
	First tumor	Second tumor	First tumor	Second tumor	
Age at diagnosis (yr) (median [range])	53.5 (29–81)		44 (27–73)	52 (31–80)	0.027
Menopausal status			0.002		
Pre‐menopausal	13 (46.4%)		77 (77.8%)	39 (39.4%)	
Post‐menopausal	15 (53.6%)		22 (22.2%)	60 (60.6%)	
Time interval between the first and second tumors (months) (median [range])	0		68 (7–342)		
Family history of breast cancer	7 (25%)		9 (9.1%)		0.047

^a^ First tumors of synchronous versus metachronous bilateral breast cancer.

The majority of patients did not have a positive family history of BC (87.4%, n = 111). However, patients with sBBC were more likely to have a positive family history of BC (25% vs. 9.1%, *p* = 0.047).

### 3.2 Pathologic characteristics of bilateral breast cancer

As shown in Table 2, the median tumor sizes of the first and second tumors in patients with mBBC were 2.65 cm and 1.5 cm, respectively. The second tumor was significantly smaller than the first tumor (*p* = 0.005). Only 1 of the 28 patients (3.6%) was histologically confirmed as having ductal carcinoma in situ (DCIS) for the first tumor. In comparison, 10 patients (35.7%) were histologically confirmed as having DCIS at the contralateral breast. In total, 27 (92.9%) patients had invasive ductal carcinoma at the first tumor, whereas only 60.7% of patients presented invasive ductal carcinoma at the second tumor. There was a statistically significant difference in the histology type between the first and second tumors in sBBC (*p* = 0.01). Furthermore, the second tumors were diagnosed at a significantly earlier tumor stage (*p* = 0.028), lymph node stage (*p* = 0.006), and TNM stage (*p* = 0.002) than the first tumors were. In patients with mBBC, there were no significant differences between the first and the second tumors with regard to the tumor size, histopathologic type, tumor stage, lymph node stage, and TNM stage.

**TABLE 2:** Pathologic characteristics of bilateral breast cancer

Characteristic	sBBC (n = 28)		mBBC (n = 99)		*p* value^a^	*p* value^b^	*p* value^c^
	First tumor	Second tumor	First tumor	Second tumor			
Tumor size (cm) (median [range])	2.65 (1.0–8.0)	1.5 (0.5–7.0)	2.5 (0.5–15)	2.0 (0.4–10)	0.005	ns	ns
Tumor stage					0.028	ns	0.028
Tis	1 (3.6)	8 (28.6)	1 (1.0)	4 (4.0)			
T1	10 (35.7)	13 (46.4)	41(41.4)	51 (51.5)			
T2	12 (42.9)	6 (21.4)	36 (36.4)	31 (31.3)			
T3	2 (7.1)	0	9 (9.1)	8 (8.1)			
T4	3 (10.7)	1 (3.6)	0	5 (5.1)			
Unknown	0	0	15 (15.2)	0			
Lymph nodal status					0.006	ns	ns
N0	12 (42.9)	24 (85.7)	48 (48.5)	56 (56.6)			
N1	5 (17.9)	3 (10.7)	17 (17.2)	17 (17.2)			
N2	7 (25.0)	0	21 (21.2)	10 (10.1)			
N3	3 (10.7)	1 (3.6)	10 (10.1)	16 (16.2)			
Unknown	1 (3.6)	0	3 (3.0)	0			
Stage					0.002	ns	ns
0	1 (3.6)	8 (28.6)	1 (1.0)	3 (3.0)			
I	6 (21.4)	11 (39.3)	27 (27.3)	38 (38.4)			
II	8 (28.6)	7 (25.0)	37 (37.4)	31 (31.3)			
III	13 (46.4)	2 (7.1)	31 (31.3)	26 (26.3)			
IV	0	0	0	1 (1.0)			
Unknown	0	0	3 (3.0)	0			
Histopathologic type					0.01	ns	ns
DCIS	1 (3.6)	10 (35.7)	3 (3.0)	7 (7.1)			
IDC	26 (92.9)	17 (60.7)	67 (67.7)	81 (81.8)			
ILC	0	1 (3.6)	11 (11.1)	7 (7.1)			
Others	1 (3.6)	0	7 (7.1)	4 (4.0)			
Unknown	0	0	11 (11.1)	0			
ER status of invasive cancers					ns	ns	ns
Negative	9 (33.3)	3 (15.0)	41 (41.8)	40 (41.7)			
Positive	18 (66.7)	17 (85.0)	55 (56.1)	56 (58.3)			
Unknown	0	0	2 (2.0)	0			
PR status of invasive cancers					ns	ns	ns
Negative	11 (40.7)	4 (22.2)	40 (40.8)	52 (54.2)			
Positive	16 (59.3)	16 (80.0)	56 (57.1)	44 (45.8)			
Unknown	0	0	2 (2.0)	0			
Lymphovascular invasion					ns	ns	ns
No	23 (85.2)	19 (95.0)	90 (91.8)	82 (86.3)			
Yes	4 (14.8)	1(5.0)	8 (8.2)	13 (13.7)			

Note

*p* value has been calculated on the known components of the variables.

Abbreviations: mBBC, metachronous bilateral breast cancer; sBBC, synchronous bilateral breast cancer; DCIS, ductal carcinoma in situ; IDC; invasive ductal carcinoma; ILC, invasive lobular carcinoma; ER, estrogen receptor; PR, progesterone receptor (PR); ns, not significant.

^a^ Synchronous first tumor versus synchronous second tumor

^b^ Metachronous first tumor versus metachronous second tumor

^c^ Synchronous first tumor versus metachronous first tumor

**FIGURE 3:**
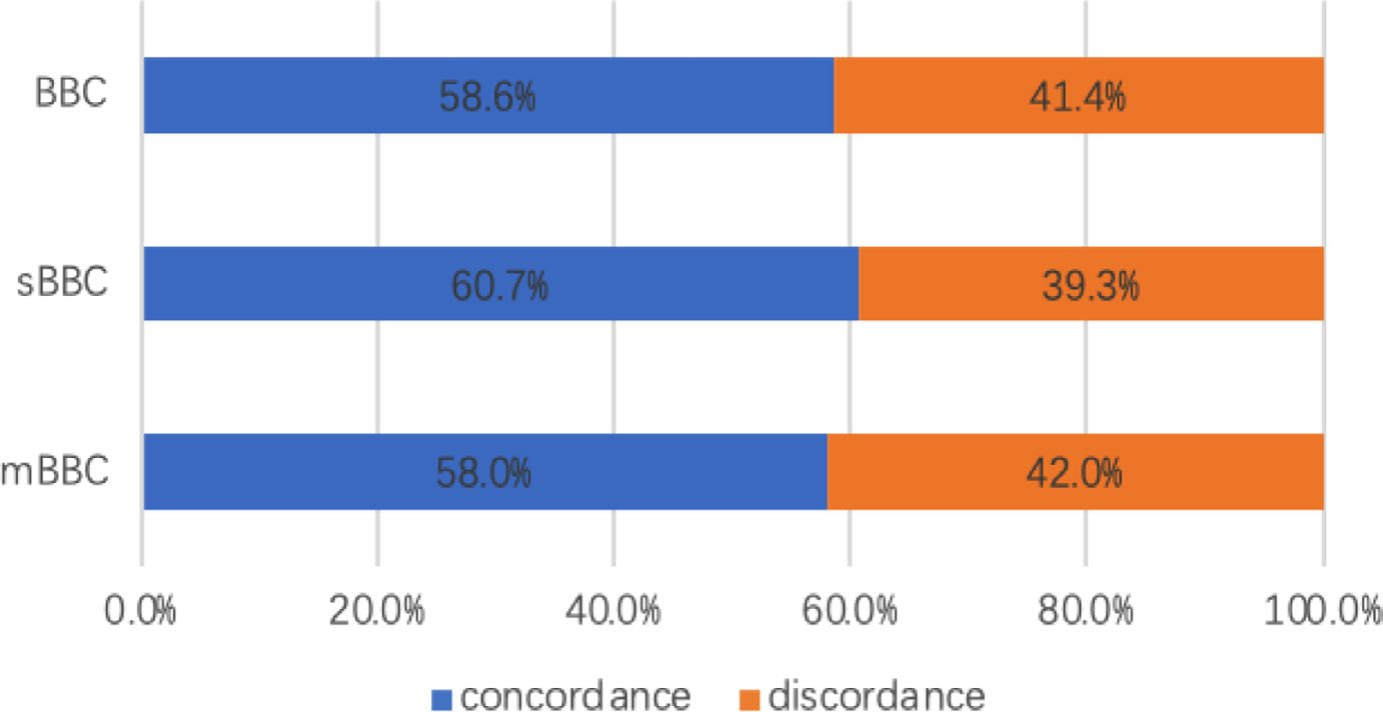
Concordance of histopathologic type in the first and second tumors for bilateral breast cancer. BBC, bilateral breast cancer; mBBC, metachronous bilateral breast cancer; sBBC, synchronous bilateral breast cancer

Between the first tumors of sBBC and mBBC, there was no statistically significant difference with regard to tumor size, lymph node stage, TNM stage, histopathologic type, ER status, PR status, or lymphovascular invasion. However, the first tumors of mBBC had a significantly earlier tumor stage than the first tumors of sBBC did (*p* = 0.028). In addition, no invasive lobular carcinomas were diagnosed in the first tumors of sBBC, whereas 11 invasive lobular carcinomas (11.1%) were diagnosed in the first tumors of mBBC (*p* > 0.05). Almost one third of patients lacked the results of HER2 status. The most common histology in BBCs was invasive ductal carcinoma. As shown in Figure 3, concordance of histopathologic type in the first and second tumors was found in 58.6% (68/116) of patients with BBC. There was no significant difference in the percentage of concordance of histopathologic type in synchronous (60.7%1, 7/28) and metachronous tumors (58.0%, 51/88).

With regard to the receptor status, there were no significant differences in ER and PR positivity of the invasive carcinomas between the first tumors of sBBC and mBBC, the first and second tumors of sBBC, and the first and second tumors of mBBC.

### 3.3 Treatment of bilateral breast cancer

Mastectomy was the most common surgical modality. There were no statistically significant differences with regard to the use of surgical modality, chemotherapy, and radiotherapy for the first tumor in patients with sBBC, compared with patients with mBBC. However, patients with sBBC were more likely to receive hormone therapy than patients with mBBC were, at the initial tumor diagnosis (82.1% vs. 61.9%, *p* = 0.045). In addition, 11 (11.1%) patients with mBBC did not undergo surgery for the contralateral tumor (Table 3). Only two patients in the sBBC group and three in the mBBC subgroup received trastuzumab therapy, respectively.

**TABLE 3:** Treatment administered in bilateral breast cancer

Treatment	sBBC (n = 28)		mBBC (n = 99)		*p* value^a^
	First tumor	Second tumor	First tumor	Second tumor	
Surgical treatment					ns
Mastectomy	26 (92.9)	24 (85.7)	88 (88.9)	74 (74.7)	
BCS	2 (7.1)	4 (14.3)	11 (11.1)	14 (14.1)	
No surgery	0	0	0	11 (11.1)	
Radiotherapy					ns
Yes	11 (39.3)	4 (14.3)	42 (42.4)	26 (26.3)	
No	17 (60.7)	24 (85.7)	57 (57.6)	73 (73.7)	
Chemotherapy					ns
Yes	22 (78.6)		80 (80.8)	79 (79.8)	
No	6 (21.4)		19 (19.2)	20 (20.2)	
Hormonal therapy					0.045
Yes	23 (82.1)		60 (61.9)	54 (54.5)	
No	5 (17.9)		37 (38.1)	45 (45.5)	

Note

*p* value has been calculated on the known components of the variables.

Abbreviations: BCS, breast‐conserving surgery; mBBC, metachronous bilateral breast cancer; ns, not significant; sBBC, synchronous bilateral breast cancer.

^a^ Synchronous first tumor versus metachronous first tumor.

### 3.4 Survival analyses

**FIGURE 4:**
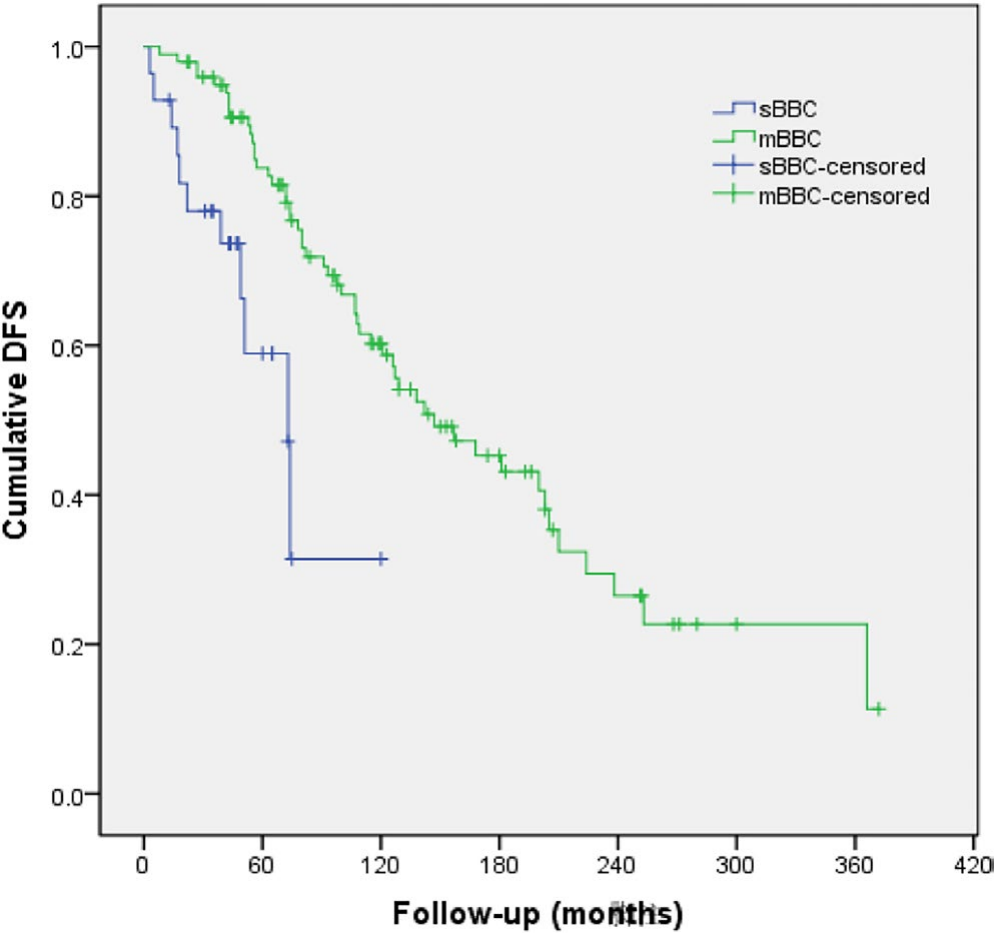
Disease‐free survival (DFS) analysis of patients with synchronous bilateral breast cancer (sBBC) versus metachronous breast cancer (mBBC)

The median duration of follow‐up was 98 (range, 12–384) months. The 5‐year DFS of sBBC and mBBC was 58.9% and 83.8%, respectively, and the 10‐year DFS was 31.4% and 60.2%, respectively. The median DFS of sBBC and mBBC was 73 months and 147 months, respectively, with a statistically significant difference (*p* = 0.001) (Figure 4).

## 4 DISCUSSION

In this study, we analyzed the proportion and outcome of BBC between 2006 and 2016 in one of the largest cancer centers of China. BBC divides into sBBC and mBBC based on the time interval between diagnoses of primary tumors in bilateral breasts. In most previous studies, the criteria of the time interval between the development of two tumors widely varied from 0 month,^11^, ^31^, ^32^ 1 month,^33^ 3 months,^30^, ^34^ 6 months,^12^, ^18^, ^35^, ^36^ to 12 months,^6^, ^37^ and the time interval of 6 months was the most frequently used. Indeed, the criteria for time intervals were artificially defined. Using different criteria of time intervals would introduce immortal bias in the analyses of sBBC and mBBC. Adopting the criteria of 6 months could reduce lead time bias and rule out the impact of adjuvant chemotherapy on the development of contralateral BC.^28^ Another meta‐analysis showed the time interval of 6 months had the least impact on survival analysis between sBBC and mBBC.^21^ In our center, it is a routine that regular ultrasound for contralateral breast every 3 months in the first 2 years after breast surgery. If found any suspicious lesion on ultrasound, breast magnetic resonance imaging (MRI) would be recommended for further assessment.^9^, ^21^, ^28^ Therefore, the time interval of 6 months was chosen as the cut‐off time to distinguish between sBBC and mBBC in our study. It is interesting to note that all of sBBC were diagnosed concurrently in our study.

The median time interval between the first and second tumors in our study was 68 months, which was longer than the time interval reported in other Asian reports^9^, ^12^ but shorter than the time interval of 80.5 months reported in a study conducted in a Western country.^4^ This short time interval of BBC in Asian patients might be attributed to the fact that the patients were enrolled 10–20 years ago and fewer patients had received adjuvant endocrine therapy, which is known to reduce the risk of contralateral BC. In our study, most of the second tumors were diagnosed within 10 years of the diagnosis of the first tumor. Therefore, annual surveillance breast imaging is essential for early detection of any contralateral BC.

The proportion of BBC in our study was 3.2%, which was similar to that reported in a previous study conducted in Southern China between 2000 and 2007.^7^ No obvious increase in the proportion of BBC was observed compared with that reported in previous studies in the Chinese population 10 years ago.^7^, ^8^, ^25^ However, in Western countries, the proportion of BBC has increased significantly in the last few decades, especially the number and proportion of sBBC.^3^, ^4^, ^5^ Patients with unilateral BC have a twofold to sixfold higher risk of developing mBBC than women in the general population do.^38^

In our study, the median age at onset of mBBC was younger than that of sBBC, which is similar to the findings of other studies conducted in China.^7^, ^25^ The median age of patients with both mBBC and sBBC was much younger in our study, which could be because women with BC in China were nearly 10 years younger than their Western counterparts at the time of diagnosis.^19^ With regard to family history, we found that patients with sBBC were more likely to have a positive family history of BC than those with mBBC were, which is similar to the findings of other studies.^20^, ^32^ However, some authors have reported no significant difference in terms of family history between sBBC and mBBC.^7^, ^10^, ^25^

In the current study, the second tumor was found to have a significantly smaller tumor size and earlier stage than that of the first tumor in sBBC. Beckman *et al*.^39^ have explained that this phenomenon might be an artifact of selecting the larger tumor as the first tumor of sBBC. However, some studies have observed these differences in both sBBC and mBBC.^6^, ^20^ Lobular histology is one of the risk factors for the appearance of BBC in the literature^39^; however, there is no consensus on whether mBBC or sBBC has a more lobular histology. Jobsen *et al*. have reported more lobular histology in mBBC,^4^ but others have reported more lobular histology in sBBC.^7^, ^18^ In this study, invasive lobular carcinomas were not diagnosed in the first tumors of sBBC, whereas 11 invasive lobular carcinomas were found in the first tumors of mBBC. With regard to the histological type, the first and second tumors were statistically different in patients with sBBC, and the most common histological type was invasive ductal carcinoma, which is in concordance with the existing literature.^15^, ^40^, ^41^ There was no significant difference in the ER and PR positivity of the invasive carcinomas between the first and second tumors, a finding similar to that of previous studies.^4^, ^12^, ^25^, ^42^

Mastectomy was the most common surgical modality used to treat both sBBC and mBBC in our study. Breast‐conserving surgery did not impair the survival of BBC.^16^ But Chinese surgeons and patients preferred to choose mastectomy because of the cautious attitude, similar to other studies in the Chinese population with primary BC.^43^, ^44^ HER2 positivity is associated with poor DFS and OS.^45^ HER2‐targeted therapy substantially improves DFS and OS in HER2‐positive early BC.^46^, ^47^ However, the results of HER2 testing were available for only two thirds of patients and very few patients received adjuvant trastuzumab therapy in this study. It was due to the expensive cost of trastuzumab beyond coverage of the healthcare system in China before September 2017. It would be unreliable to further analyze HER2 status and HER2‐targeted therapy between the sBBC and mBBC subgroups. Hormone therapy application rates were higher in the sBBC group than that in the mBBC group (82.1% vs. 61.9%, *p* = 0.045) despite no significant differences in ER and PR positivity of the invasive carcinomas between the first tumors of sBBC and mBBC in our study. This implied that patients with sBBC had better compliance to hormone therapy than patients with mBBC. Although adjuvant hormone therapy could have an effective favorable impact on survival,^48^ sBBC still showed a significantly poorer DFS compared with mBBC in our study; the median DFS was 73 and 147 months for sBBC and mBBC, respectively. These results are partly consistent with those of the study conducted by Kheirelseid *et al*., which reported a median DFS of 52 months for sBBC and 148 months for mBBC, with significant difference.^6^ Previous studies have reported inconsistent results regarding the prognosis of sBBC and mBBC. Some studies have reported similar outcomes between sBBC and mBBC,^15^, ^18^, ^49^ but other studies have reported a poorer survival of patients with mBBC than those with sBBC.^25^, ^39^

This study has several limitations. First, this was a single‐center retrospective analysis, and there might have been a bias in patient selection. Second, the sample size was small, especially as the analysis of family history was based on seven patients with sBBC and nine patients with mBBC, and the *p* value was marginally significant. Third, because the healthcare system did not cover the HER2‐targeted therapy before 2017 in China, there was lack of enough information on HER2 status and only a tiny minority of patients received adjuvant trastuzumab therapy in this study. We were not able to compare HER2 status and HER2‐targeted therapy between sBBC and mBBC. Further studies will be needed for epidemiologic incidence of BBC in China and underlying molecular mechanism of BBC, especially sBBC.

## 5 CONCLUSION

BBC is a rare disease entity in the Chinese population, and the proportion of sBBC is lower than that of mBBC. sBBC and mBBC might be different genetical subgroups of BC. Patients with sBBC show a significantly greater prevalence of a family history of BC and poorer prognosis compared with patients with mBBC. Most of the second tumors in mBBC were diagnosed within 10 years of the diagnosis of the first tumor.

## AUTHOR CONTRIBUTION STATEMENT

HJ and HL designed this study. HJ, RZ, RR, and HL analyzed and interpreted the data. HJ drafted the manuscript and performed statistical analysis. JZ, YL, XG, YC, BS, YY, XL, GS, and LD involved in patient care, follow‐up, as well as manuscript review and revision. HJ and KL contributed to collect data. XL contributed to statistical analysis. HL supervised this study and revised the manuscript. All authors read and approved the final manuscript.

## CONFLICT OF INTEREST

The authors declare that they have no conflict of interest.

## ETHICAL STATEMENT

This study was approved by the Ethics Committee of Peking University Cancer Hospital (Approval ID: 2020YJZ82), which waived the requirement for patient signed informed consent owing to the retrospective nature of the study. And all procedures performed in this study involving human participants were in accordance with the ethical standards of the Ethics Committee of Peking University Cancer Hospital and with the declaration of Helsinki.

## Data Availability

The data that support the findings of this study are available from the corresponding author upon reasonable request.
